# What can motivate Lady Health Workers in Pakistan to engage more actively in tuberculosis case-finding?

**DOI:** 10.1186/s12889-019-7326-8

**Published:** 2019-07-25

**Authors:** Mishal S. Khan, Nelofar Mehboob, Afifah Rahman-Shepherd, Farah Naureen, Aamna Rashid, Naveed Buzdar, Muhammad Ishaq

**Affiliations:** 1 London School of Hygiene & Tropical Medicine, 15-17 Tavistock Place, London, WC1H 9SH UK; 2 Mercy Corps Pakistan, Islamabad, Pakistan; 3 Centre on Global Health Security, Chatham House, 10 St James’s Square, London, SW1Y 4LE UK

**Keywords:** Community health worker, Pakistan, Financial incentive, Tuberculosis

## Abstract

**Background:**

Many interventions to motivate community health workers to perform better rely on financial incentives, even though it is not clear that monetary gain is the main motivational driver. In Pakistan, Lady Health Workers (LHW) are responsible for delivering community level primary healthcare, focusing on rural and urban slum populations. There is interest in introducing large-scale interventions to motivate LHW to be more actively involved in improving tuberculosis case-finding, which is low in Pakistan.

**Methods:**

Our study investigated how to most effectively motivate LHW to engage more actively in tuberculosis case-finding. The study was embedded within a pilot intervention that provided financial and other incentives to LHW who refer the highest number of tuberculosis cases in three districts in Sindh province. We conducted semi-structured interviews with 20 LHW and 12 health programme managers and analysed these using a framework categorising internal and external sources of motivation.

**Results:**

Internal drivers of motivation, such as religious rewards and social recognition, were salient in our study setting. While monetary gain was identified as a motivator by all interviewees, programme managers expressed concerns about financial sustainability, and LHW indicated that financial incentives were less important than other sources of motivation. LHW emphasised that they typically used financial incentives provided to cover patient transport costs to health facilities, and therefore financial incentives were usually not perceived as rewards for their performance.

**Conclusions:**

This study indicated that interventions in addition to, or instead of, financial incentives could be used to increase LHW engagement in tuberculosis case-finding. Our finding about the strong role of internal motivation (intrinsic, religious) in Pakistan suggests that developing context-specific strategies that tap into internal motivation could allow infectious disease control programmes to improve engagement of community health workers without being dependent on funding for financial incentives.

## Background

Performance of the health workforce is one of the weakest and most neglected components of the health system in low and middle-income countries (LMIC) [1] . A well-functioning community health worker network has been critical to strengthening the health system in several LMIC by addressing shortages in the number and distribution of trained healthcare providers [2–4]. A distinguishing feature of community health workers is that they typically live in and belong to the community they serve, and usually have basic training to provide limited medical and public health services, including neonatal care, immunisation, health education and case-finding for infectious diseases [5]. In their review of community health worker programmes worldwide, Perry and colleagues highlighted that studies in numerous settings have documented that community health worker involvement in service delivery can enhance public health outcomes, including improved management of infectious diseases [6]. Efforts are thus being made to find sustainable strategies to engage community health workers in control of infectious diseases [3, 7, 8], of which tuberculosis (TB) is the number one cause of death globally [9].

Many interventions designed to motivate community health workers to do more or better work have relied heavily on provision of financial incentives contingent upon achieving specific targets (performance-based incentives [PBIs]). There has been significant investment in PBI projects since 2008, including US$ 420 million from the Health Results Innovation Trust Fund and a further US$ 2.4 billion from the International Development Association [10]. As a result of this large investment, supply-side PBIs have been implemented and scaled up in many countries, especially in Africa [11].

However, evidence from social science research questions the assumption that financial gains are the main source of motivation for health workers. Distinctions have been made between internal and external sources of motivation and subsets of drivers of motivation within these two categories (Table 1) [12–14]. In the context of community health workers, external motivation does not come from satisfaction related to the activity itself, but instead stems from material rewards or possible consequences. These consequences could include tangible monetary or non-monetary rewards or social recognition. In contrast, internal motivation does not rely on external recognition of performance. It can be driven, for example, by the satisfaction derived from providing care to patients (intrinsic) or from the religious reward envisaged as resulting from the activities [14, 15].

**Table 1:** Summary of conceptulisation of different sources of motivation

External	Monetary rewards	Individual is driven by the desire to receive financial rewards
	Non-monetary material rewards	Individual is driven by the desire to receive non-monetary rewards that have a financial value (such as food packages or health insurance)
	Social	Individual is motivated by recognition from peers, community members or managers
Internal	Religious/moral	Individual believes that the activity is a religious or moral duty, or links activity to non-material rewards from a religious perspective
	Intrinsic	Individual derives spontaneous satisfaction from the activity itself

Apart from the diverse factors potentially influencing community health workers’ motivation, achieving an impact with PBI schemes requires support from and capacity in local institutions. Contextual factors – such as weak local accountability mechanisms, limited bureaucratic capacities, gaps in information systems, and wider issues of organisational culture – have been shown to affect the implementation of financial incentive schemes [16].

Consistent with the concerns raised about the use of PBI schemes discussed above, empirical evidence shows that financial incentives alone do not maximise the worker’s motivation potential; a large systematic review of interventions designed to influence the performance of community health workers in LMIC found that improved performance was associated with a mix of incentives, including frequent supervision, continuous training, community involvement and strong coordination and communication between community health workers and health professionals [17]. Before further investment in large-scale PBI schemes in LMIC, more research is required to understand how combinations of incentives (financial and otherwise) should be balanced in order to optimally motivate community health workers.

In order to inform the design of future programmes that aim to incentivise LHW to have greater involvement in TB case finding, this study investigates the relative importance of different sources of LHW motivation in one high TB-burden country – Pakistan - and explores the level of programme manager support for the scale-up of PBIs to improve LHW engagement in TB case-detection.

## Methods

### Study setting

Pakistan is a populous country of over 220 million. Since health is devolved, each province is responsible for healthcare provision to its population, and the National TB Programme (NTP) therefore operates through Provincial TB Programmes. Like many LMIC, secondary and tertiary public hospitals are typically overcrowded, and primary care services are weak and underutilised [18]. Primary care in Pakistan is delivered through Basic Health Units and Rural Health Centres – which are small healthcare facilities - and through Lady Health Workers (LHW) who go operate from their houses and are based within the community they serve. The Pakistani Government initiated the LHW programme in 1994 and there are now more than 100,000 LHW deployed across the country [19]. This cadre of community health workers plays a particularly important role in providing primary healthcare in rural areas and urban slums in Pakistan, covering 60% of the population [20]. Each LHW is attached to a government health facility and is provided with training, supervision, medical supplies, and a small allowance of approximately US$ 300 per year [21]. LHW are usually responsible for approximately 100–200 households, and although they have mainly focused on maternal and child health, there is an interest in engaging LHW in improving community level infectious disease control [22].

TB remains one of the most critical infectious diseases to tackle in Pakistan, contributing to substantial mortality and morbidity [23]. Pakistan has the fifth highest burden of TB in the world, with over half a million new cases detected every year [24, 25]. An enhanced role of LHW may be particularly important as there is an urgent need for improved referral of TB patients in rural communities for early diagnosis. Early detection and treatment of TB is lacking, and it is estimated that almost 40% of TB cases are not able to access free treatment offered by the government’s National TB Control Programme [24].

Improving early case finding and appropriate treatment in Pakistan is critical because delays in diagnosis result in ongoing transmission and increase the risk of emergence of drug resistance [23]. Over the past decade, several approaches to improve TB case detection have been piloted in Pakistan, including chest x-ray camps and improved coordination with for-profit healthcare providers [26, 27]. Although TB is already listed as part of LHW broad range of duties, TB care is to be provided in addition to core services on maternal and child health and family planning, alongside malaria control, polio vaccination drives, and treatment minor ailments [25]. There is interest in better engaging LHW in improving TB case finding among the poorest patients living in rural areas because LHW are the cadre of health workers most suited to providing health services to communities and individuals that are difficult to reach, acting as a link between communities and health facilities that can provide TB diagnostic services [28].

The majority of funding for TB control in Pakistan is provided by The Global Fund to Fight AIDS TB and Malaria, and the non-governmental organisation Mercy Corps (MC) is a primary recipient of funding to implement TB control activities in Pakistan. During 2018, MC piloted an intervention in which LHW received monetary incentives when they referred a patient to a government health facility who was diagnosed with TB. Additionally, award ceremonies were introduced, during which LHW who had referred the highest number of TB patients for testing in each district are presented with shields by their supervisors.

The introduction of this pilot reward scheme, which was rolled out in Ghotki, Sanghar and Umerkot districts in Sindh province, Pakistan, provided an opportunity to conduct our study in these.

### Study participants

We sought to understand the perspectives of representatives of two key stakeholder groups: LHW engaged in TB case finding by MC (recipients of any reward package) and health programme managers at the district level (who would be responsible for integrating any new reward schemes into the existing health system).

We used purposive sampling to identify potential interviewees. All interviewees we approached agreed to participate. Access to relevant district-level programme managers was facilitated by the formation of a Provincial Inter-Program Coordination Committee (PIPC) to oversee the pilot PBI intervention. The PIPC consisted of representatives of the government TB and LHW programmes. Members of the PIPC were approached for interviews and requested to identify other key decision-makers at the district level. Using this process we identified 12 health programme managers to interview, including individuals who were responsible for running TB or LHW programmes in the study districts, or for directly supervising LHW.

To facilitate purposive selection of 20 LHW, we obtained a list of all LHW working on the MC TB control pilot programme in the study districts. From this list we selected individuals to ensure that LHW with different durations of work experience were represented, all three districts were represented and to include LHW who had and had not received any rewards from MC for TB case-finding (capturing a range of individual experiences with the incentive programme).

### Data collection and analysis

Following written informed consent, face-to-face interviews lasting between 30 and 40 min were conducted in Urdu or Sindhi at a location selected by the interviewee within the interviewee’s home or work district. A female Pakistani researcher (NM) with expertise in qualitative methods and familiarity with the local context conducted one-to-one, private interviews. A pre-piloted, semi-structured interview guide was used to explore interviewees’ perspectives. The topic guide covered the following topics: reasons for joining and staying with the LHW programme, different sources of motivation, views on monetary and non-monetary rewards, and barriers or facilitators to their work as LHW. Open ended questions were asked and interviewees were given time to express their own opinions and ideas. In many cases their responses shaped the flow of the interviews. Interviews were recorded and transcribed verbatim.

We used a combination of deductive and inductive thematic analysis, based on an interpretive approach [29]. Each anonymised transcript was coded line by line in NVivo (v12). One researcher (MSK) conducted a first round of deductive analysis using sources of motivation identified through our literature review and summarised in Table 1 to guide coding: Social; Monetary rewards; Non-monetary rewards; Religious; Intrinsic. Within each of these categories of sources of motivation, an inductive approach was used by two researchers (MSK, NM) to identify salient themes.

Ethical approval was granted by the Ethics Committee of International Research Force (Pakistan) and written informed consent was provided by each interviewee.

## Results

The 20 LHW we interviewed were aged between 30 and 45 and worked in Ghotki (4), Sanghar (10), Umerkot (6). Of the 12 programme managers we interviewed, three were female, and all were aged between 35 and 45. We present our findings about external and internal sources of LHW motivation sequentially, illustrated with excerpts from the interviews in Tables 2 and 3. We then summarised recurring themes related to scaling up and sustainability of LHW reward schemes to enhance TB case-finding at community level.

**Table 2:** Illustrative excerpts from interviews on external sources of motivation

Monetary rewards	•The reason of this job is to support my family. Due to some reason, my husband is not working and stops me to do so, too. He is addict, use foul language, I am the sole earner of family for 20 years (LHW G) •This is a basic principle that individual work for reward or money, and this is a source of encouragement, you can inspire with two methods, as these days’ people have financial crisis, therefore, monetary incentives attract more. (LHW G) •In my opinion, whoever paid well, will perform well, Money will act as petrol and she will run fast for her work. If will not get any compensation, she will say there are ten more who are sitting and doing nothing, why should I go? (Programme manager G) •The monetary incentive given for TB is adequate, even if it’s not given, this is our duty and we need to do it at any cost. (LHW S) •The reward of rupees 1000 is given per TB case is being utilized for all the transport expenses of the patient till his last check-up. LHW belong to poor families, so if we have money only then we can help our patients. (LHW S) •The amount of Rs. 1000 should be given to us because we bring patients from far flung areas, when they reach at centre, sometimes no electricity, staff is on leave, then we have to bring them again, so almost Rs. 500 is spend on commuting, therefore, the amount is very less and it should be increased. (LHW U) •As per my observation, if there are incentives, LHW remains motivated and they achieve their targets well, but if they don’t receive any incentives, they will work just like that without any efforts to achieve her target. They won’t bother to send referrals, so without incentives, there is no desire to work. (Programme manager U)
Non-monetary material rewards	•Health workers should get food package as per their economic conditions, also, free medical care must be provided (LHW G) •In my opinion, providing other facilities instead of money is better for LHW. Compensating with money is not a bad thing, as it is reward of their hard work, but, offering health and other facilities will be more beneficial. (Programme manager G) •We are tired of borrowing money from people, therefore, we want that we should be given food packages on our good performance or at least we should be paid regularly, so that we don’t need anything else. (LHW S) •There are field workers who demands for food packages on their good performance, but it seems inappropriate, though there must be free medical facility (LHW S) •The financial status of most of the LHW is not that good, so if they are given a food package on their good performance, this will be beneficial and a source of motivation for them. (Programme manager S) •If we get free medical facility, it would be better (than monetary awards) because sickness makes us worry. (LHW U) •In return of our work and best performance, if we get free medical facility we will be happy because good health is important. If we are ill, how can we work? If we get proper medicine and access to the doctor for check-up, then it will be a good incentive, whereas food packages are not that important for us because, if we get salary on time, we can buy our own food ration. (LHW U) •If other companies are providing incentives on the basis of performance then we should also we should also get medical facility. God gives us food; we can arrange our own food but free medical should be given. (LHW U)
Social	•Look, wherever you are working, appreciation is important, one is working hard and if there is no appreciation then consider it as incomplete. (Programme manager G) •There was an event for girls who performed well, I was so delighted to receive an award. It gives us encouragement to see that we get acknowledged among people, there is someone, who cares about our efforts. (LHW G) •Few workers were given shields as a token of appreciation, but I was not given one, since I had less TB patients. I hope to get a shield next time. (LHW S) •In my opinion, getting shield among everyone is a matter of pride. Money comes and spend but the impact of recognition in terms of shield remains for longer period (LHW S) •In the seminar I was honoured and when in front of everyone bestowed the award, I showed it on my return to the village that I got this award for the work I have done, so it made me happy. (LHW U) •Elders in the community also gives us respect, and take my advice on health matters, they come to me first. (LHW G) •People in the area love us and respect us because we do work and visit each home. (LHW G) •I never thought of leaving this job, I continue it because I get respect from others. (LHW G) •People give us extra importance and respect us. Whenever, I visit the household they bring stuff to eat, despite refusing for it. They say you are our ‘baji’ who came to help us, then should have some tea or soft drink. (LHW S) •I am proud of working as LHW. I can be helpful for others; women share their private matters with us, which they can’t do with other people. (LHW S) •Community people appreciate our efforts and asks, when we will meet them and visit their houses, they give us more respect. They ask about us, when our visit got delayed or take more days. They trust us and listen to our advice attentively, it makes us happy, particularly, when they follow, what we told them to do. (LHW U) •Community is recognizing LHW work because they realize their performance in the health matters. (Programme manager U)

**Table 3:** Illustrative excerpts from interviews on internal sources of motivation

Intrinsic	•I enjoy helping people by increasing their information about illnesses and how to tackle them. I want to help them. (LHW G) •If someone get better from our efforts, it gives us happiness (LHW G) •We don’t have work related problems, instead we feel happy when we help someone regarding any disease because we serve people as a humanitarian work. (LHW S) •I personally feel that even if I don’t get paid for TB work, I’ll still give services as humanitarian work.. (LHW S) •So money is secondary, I joined this profession with the passion to serve the community. (LHW S) •It is not like every worker works for money, there are others who accept to work without it and work whole heartedly. (Programme manager U) •I am very happy with the work as I came to know how people are living in their socio economic condition, so I feel happy to provide them services. (LHW U)
Religious/Moral	•The livelihood is from God; He provides it on my doorstep. I am so proud that He choose me for this (LHW G) •The amount given for TB case is sufficient for me, just God bestow His blessings on it. Patient will recover, his/her life doesn’t buy from money. God gave us a chance to share information and about free health facility to poor patients through this programme. (LHW G) •I like my work because we serve poor and deprived people who doesn’t know about different diseases and we provide them awareness about it. I feel blessed as God chosen me to serve his people. (LHW G) •We are working continuously and God is rewarding us for our efforts (LHW G) •This job is not only a source of our income and our need, but, also we are getting its religious reward. (LHW S) •There are some very old women who cannot even sit or walk properly and when they pray for us or caress our foreheads, we find peace in our hearts. We get a different kind of happiness. (LHW S) •LHW are really performing their duties and responsibilities well. They consider it as a humanitarian work and feel happy to spread awareness to the community people (Programme manager S) •We get Rs. 1000 on per positive case in TB program, this amount is sufficient, we should consider that if someone’s life is saved it’s a big achievement. Money is just like the dirt on your hands, which will not remain forever. So, if a human’s life is saved, it is very important to us (LHW U) •And if, someone’s live saves because of our efforts, it a Sawaab. Salary is a separate case, whosoever work, he/she can get, but, this is matter of Sawaab (LHW U)

### External sources of motivation

Monetary rewards were clearly considered an important source of motivation by the majority of LHW and programme managers interviewed. However, a consistent finding in all three districts was that financial incentives were judged to only be part of LHW motivation and most did not mention financial gain as their main driver.

Monetary income was the most commonly mentioned reason to explain initial interest in working as an LHW, but less so in relation to ongoing motivation. Approximately half of LHW explained that they started working as an LHW because there was insufficient household income from their husband or father. Those who mentioned starting work as an LHW to earn financial benefits all went on to say that they have continued in the job due to other (non-financial) motivating factors.

However, it was apparent that support from family members (husbands, mothers-in-law, brothers) was contingent on the financial rewards of the job, and that such support is important for LHW to continue working:

*My family supports me because this job helps me for my children’s education, so they like it. (LHW U)*

As family member support was highlighted frequently as an important consideration by LHW, financial benefit appears to have a direct and indirect (via family member support) impact on motivation of LHW.

An important finding emerging from our analysis is that specific financial incentives provided for TB case-finding were often perceived by LHW not as rewards for their efforts but instead were viewed as payments to cover patient transport to health facilities and ancillary medical checks (which otherwise LHW have to try to pay themselves). This was mentioned by LHW on 12 different occasions, for example:

*The reward of rupees 1000 given per TB case is being utilised for all the transport expenses of the patient till his last check-up. LHW belong to poor families, so if we have money only then can we help our patients. (LHW S)*

There were contrasting views about non-monetary material rewards such as health insurance and food packages. Some LHW felt that food packages would be appreciated as a reward for good work, primarily because of salary fluctuations. Others believed that the food packages should be given to patients as incentives to initiate TB treatment or to help them respond more effectively to medication. While not stated explicitly, when mentioning that patients rather than LHW should be given food packages, one LHW implied that LHW should be paid enough salary to buy their own food whereas food ‘handouts’ should be reserved for those with a lower socioeconomic status.

Overall, food packages were often perceived to be secondary to salary (paid on time) and PBIs. Health insurance was considered by most LHW as a more attractive non-monetary reward than food packages, and two programme managers also encouraged consideration of non-financial rewards such as health insurance:

*If we get proper medicine and access to the doctor for check up then it will be a good incentive, whereas food packages are not that important for us because, if we get salary on time, we can buy our own food ration. (LHW U)*

The final external driver of motivation we identified, social recognition, indicated that both appreciation from the community and accolades in front of peers or supervisors were strong motivational drivers, with some LHW indicating that this driver is more important than monetary rewards. Comments about award ceremonies were positive, both from LHW who had received awards and from those who had not been given an award but were working toward one. It was apparent that LHW felt that their work on TB had not been recognised earlier and the award ceremonies introduced by MC were a welcome change. Programme managers also consistently agreed that award ceremonies were important as motivators:

*In my opinion, getting a shield among everyone is a matter of pride. Money comes and is spent but the impact of recognition in terms of a shield remains for longer period (LHW S)*

The LHW position in the community, the special respect given to them by elders and people who they visit was described frequently as a motivation to stay engaged with the job. Several LHW also explained that they appreciate the trusted position they hold, as indicated to them by community members (especially women) confiding in them:

*I am proud of working as LHW. I can be helpful for others; women share their private matters with us, which they can’t do with other people. (LHW S)*

*I never thought of leaving this job, I continue it because I get respect from others. (LHW G)*

*The community is recognizing LHW’s work because they realise their performance in the health matters. (Programme manager U)*

### Internal sources of motivation

As summarised in Table 3, internal motivation – unrelated to any material rewards or social recognition – was discussed frequently in interviews. A supervisor responsible for management of all LHW in her district compared motivation levels across her supervisees and identified two factors – level of LHW education and genuine ‘fondness’ for the job – as the main determinants of differences between the best and worst performers. The ‘joy’ from caring for or helping patients was mentioned by the majority of LHW as a spontaneous source of motivation to conduct their daily job-related activities. The motivation derived from caring for others was often linked to moral or religious values too; service to humanity was talked about in terms of spontaneous joy or religious reward that comes from saving a life.

Some LHW mentioned additional factors that led to intrinsic motivation for conducting their job, including the chance to socialise with other women in the community and learn new things.

A significant finding from our analysis is that the desire to ‘serve humanity’ and be rewarded by God was a strong driver of motivation for LHW. The word ‘sawaab’ was used frequently to describe the religious reward that motivates LHW. Working to gain rewards from God was among the most frequently mentioned factors driving LHW to work hard irrespective of payment received. The benefits from prayers or *sawaab* were described by some LHW as being more valuable to them than money:

*Everyone has to leave this world, so if we are doing some work we should always do it properly because for that we get prayers from people. I think money and prayers are two different things; where prayers work, money does not (LHW S)*

Although not all LHW directly compared monetary rewards with internal sources of motivation, when they did they expressed a strong sentiment that financial gain was a secondary consideration:

*So money is secondary, I joined this profession with the passion to serve the community. (LHW S)*

We get 1000 rupees per positive case in the TB program, this amount is sufficient. We should consider that if someone’s life is saved it’s a big achievement. Money is just like the dirt on your hands, which will not remain forever. So, if a human’s life is saved, it is very important to us (LHW U).

### Opportunities and challenges for large-scale engagement of LHW in TB case-finding

District health programme managers raised specific concerns about scale-up of monetary rewards to LHW for TB case-finding, and our analysis indicated that there are opportunities to motivate LHW without use of monetary incentives.

Programme manager’s concerns or hesitations about use of monetary rewards to engage LHW in TB case-finding included the following: distraction from their key role, which is maternal and child care; building a culture among LHW of reliance on monetary incentives to conduct duties that are part of their core role; and financial sustainability if the incentive programme needs to be funded in future using government resources:

*I can’t support anything monetary because of sustainability issue, what happens if the programme ends tomorrow? (Programme manager U)*

Addressing programme managers’ concerns with reliance on financial incentives to improve TB case-finding by LHW, some LHW put forward specific suggestions favouring alternative strategies. A recurring theme (mentioned 15 times by LHW across all districts) was that provision of financial or other support for patients’ transportation is critical to improving referral of TB patients by LHW. LHW explained that poor patients who are referred by them for TB testing do not have enough money to reach the health facility or pay for medical tests. LHW therefore face challenges in convincing patients to visit health facilities or take on the financial burden to ensure that patients with TB symptoms are tested. Related to this point, a small number of LHW strongly urged programme planners to provide payments to LHW not only for patients who test positive for TB, but also for every symptomatic patient for whom they facilitate transport to the health facility. Here the key point made was that LHW often have to cover transport costs for all patients with TB symptoms, and if a patient is not diagnosed with TB the LHW is not reimbursed:

*...other than positive case at least 150 rupees should be given for negative cases, it would be much appreciated. The reason is our commuting cost. Now, who is patient and who is not, we don’t know, we only refer them. The case gets confirmed after the test, but our cost occurs already, therefore, at least rickshaw fare should be given (LHW U)*

Finally, a frequently mentioned problem, and opportunity for enhancing LHW motivation, is the issue of delayed salary payments. Although other aspects of the job, such as flexible timing and being able to work in the local area, were described in positive terms by LHW, long and regular delays in receiving salary payments were identified as being detrimental to LHW performance by both LHW and programme managers:

*We don’t have work problem, but if I share honestly, we don’t get our monthly salary regularly and it is very disturbing. If we get our salary on time, we enjoy our work and will be less worried about home (LHW G)*

*LHW main issue is her salary which she never gets on time…If they get timely salary payments along with better incentives, then their work will be better ... (Programme manager G)*

## Discussion

Our study indicates that although monetary income plays a role in motivating LHW to conduct their duties, particularly when the income is essential to financially support their families, other external and internal sources of motivation are important to consider when designing programmes to engage LHW in TB case-finding activities. For example, Pakistani LHW accounts of the financial burden they face when supporting transport costs for patients with TB symptoms highlight the immense challenges around physical access to health facilities in rural areas where health systems are under-resourced; these access to healthcare issues will not be overcome by financial incentives to LHW alone. The overall negative impact on LHW motivation from delays in salary payments was also emphasised by both LHW and programme managers, and this could counteract the positive impact of incentive payments.

A number of common points emerge from the combined analysis of LHW and programme manager interviews. First, we found that internal drivers of motivation – unrelated to any third party’s acknowledgement of the LHW performance – were salient in our study setting and have not been taken into account adequately when designing performance management schemes. Motivation to serve humanity and save lives by supporting TB diagnosis was frequently linked to LHW’s religious values and thoughts about future rewards from God. Internal motivation linked to intrinsic joy or religious reward for helping TB patients appeared to be a stronger driver than monetary rewards. It was also highlighted through interviews with programme managers that focusing on increasing motivation without relying heavily on monetary incentives is a strategy that they consider more financially sustainable. The role of religious beliefs as a strong motivating factor for health workers has been identified in African settings as well, such as Uganda, Ethiopia and Rwanda, although this is not a widely investigated area [30, 31].

Second, in terms of external drivers of motivation, social recognition in the form of performance-related rewards and respect from the community was an important motivator. A mixed methods analysis in neighbouring India similarly found that community health workers were strongly motivated by the desire to gain recognition and respect from their communities and families, in addition to a sense of responsibility towards their communities [32]. A survey-based study of job-related motivation of primary healthcare workers in Cambodia found that they are “altruistic and value community services, and good community and peer relations”, concluding that improving motivation does not always require monetary incentives [33]. Thus, there is evidence from several studies using different methodologies, including our qualitative study in Pakistan, which indicates that community health workers’ motivation can be increased through non-monetary incentives. A striking finding in our study was that many LHW did not perceive the PBIs they received to be rewards for TB case-finding, but rather considered this extra income as money to support transport for TB patients to health facilities (which LHW would otherwise have to pay for themselves).

Given the qualitative nature of this study and the context-specific nature of motivation, we appreciate that we cannot generalise these findings to other countries or even other provinces in a large country such as Pakistan. Indeed the balance between drivers of motivation or barriers to TB case-detection varies in different parts of Pakistan and it is widely agreed that careful consideration of context is required when developing performance management schemes [34–36]. In terms of study limitations, we also acknowledge that logistical and time constraints meant that it was not possible to receive participant verification of our results. The study also faced a risk for social desirability bias as interviewees may have downplayed the impact of monetary reward to avoid coming across as avaricious.

Despite these limitations, the in-depth analysis of a sample of LHW and health programme managers from three rural districts in Pakistan suggests that studying drivers of motivations using a clearly defined analytical framework can be informative for programme planning. Considering the limited evidence on harnessing intrinsic motivation of community health workers, a randomised trial or other large-scale evaluation that compares the impact on TB case-finding by LHW of monetary incentives versus strategies that target intrinsic sources of motivation (for example, awards given at community functions, appreciation by religious leaders) may be useful in informing strategies of service delivery organisations. It may also be valuable to consider the impact on equity of access to health services when community health workers are better engaged in infectious disease control; for example, it is envisaged that LHW involved in TB control may improve case-finding among women in Pakistan, but this has not been assessed.

## Conclusions

Embedded within a nascent PBI scheme in Pakistan, our study identified considerations that could be useful in shaping programmes to engage community health workers in TB case-finding. Specifically, our results indicate that interventions in addition to, or instead of, financial incentives could be used to increase LHW engagement in TB case-finding. Our finding about the strong role of internal motivation (intrinsic, religious) points to potential strategies to address concerns about sustaining financial reward schemes without external funding. In contexts where communities have strong religious beliefs, programmes could harness community health workers’ motivation to gain religious rewards without creating a dependency on financial incentives. Developing context-specific strategies that tap into internal motivation could allow programmes that do not have the resources to fund PBI to improve engagement of community health workers in infectious disease control programmes.

## Data Availability

The datasets analysed during the current study are available from the corresponding author on reasonable request to any scientist wishing to use them for non-commercial purposes, without breaching participant confidentiality and study ethical approval conditions.

## Abbreviations

LHW: Lady Health Workers
LMIC: Low and middle income countries
MC: Mercy Corps
PBI: Performance-based incentives
PIPC: Provincial Inter-program Coordination Committee
TB: Tuberculosis
